# Enhancing PRRT Outcome Prediction in Neuroendocrine Tumors: Aggregated Multi-Lesion PET Radiomics Incorporating Inter-Tumor Heterogeneity

**DOI:** 10.3390/cancers17233887

**Published:** 2025-12-04

**Authors:** Maziar Sabouri, Ghasem Hajianfar, Omid Gharibi, Alireza Rafiei Sardouei, Yusuf Menda, Ayca Dundar, Camila Gadens Zamboni, Sanchay Jain, Marc Kruzer, Habib Zaidi, Fereshteh Yousefirizi, Arman Rahmim, Ahmad Shariftabrizi

**Affiliations:** 1 Department of Physics and Astronomy, University of British Columbia, Vancouver, BC V6T 1Z4, Canada; 2 Department of Basic and Translational Research, BC Cancer Research Institute, Vancouver, BC V5Z 1L3, Canada; 3 Division of Nuclear Medicine and Molecular Imaging, Geneva University Hospital, 1205 Geneva, Switzerland; 4 Department of Electrical and Computer Engineering, University of British Columbia, Vancouver, BC V6T 1Z4, Canada; 5 Division of Nuclear Medicine, Department of Radiology, University of Iowa Carver College of Medicine, Iowa City, IA 52242, USA; 6 MIM Software Inc., Cleveland, OH 44122, USA; 7 Department of Radiology, University of British Columbia, Vancouver, BC V6T 1Z4, Canada

**Keywords:** PET, radiomics, feature aggregation, PRRT, Neuroendocrine Tumors

## Abstract

Introduction: Peptide Receptor Radionuclide Therapy (PRRT) with [^177^Lu]Lu-DOTA-TATE is effective in treating advanced Neuroendocrine Tumors (NETs), yet predicting individual response in this treatment remains a challenge due to inter-lesion heterogeneity. There is a lack of standardized, effective methods for using multi-lesion radiomics to predict progression and Time to Progression (TTP) in PRRT-treated patients. This study evaluated how aggregating radiomic features from multiple PET-identified lesions can be used to predict disease progression (event [progression and death] vs. event-free) and TTP. Methods: Eighty-one NETs patients with multiple lesions underwent pre-treatment PET/CT imaging. Lesions were segmented and ranked by minimum Standard Uptake Value (SUV_min_) (both descending and ascending), SUV_mean_, SUV_max_, and volume (descending). From each sorting, the top one, three, and five lesions were selected. For the selected lesions, radiomic features were extracted (using the Pyradiomics library) and lesion aggregation was performed using stacked vs. statistical methods. Eight classification models along with three feature selection methods were used to predict progression, and five survival models and three feature selection methods were used to predict TTP under a nested cross-validation framework. Results: The overall appraisal showed that sorting lesions based on SUV_min_ (descending) yields better classification performance in progression prediction. This is in addition to the fact that aggregating features extracted from all the lesions, as well as the top five lesions sorted by SUV_mean_, lead to the highest overall performance in TTP prediction. The individual appraisal in progression prediction models trained on the single top lesion sorted by SUV_min_ (descending) showed the highest recall and specificity despite data imbalance. The best-performing model was the Logistic Regression (LR) classifier with Recursive Feature Elimination (RFE) (recall: 0.75, specificity: 0.77). In TTP prediction, the highest concordance index was obtained using a Random Survival Forest (RSF) trained on statistically aggregated features from the top five lesions ranked by SUV_mean_, selected via Univariate C-Index (UCI) (C-index = 0.68). Across both tasks, features from the Gray Level Size Zone Matrix (GLSZM) family were consistently among the most predictive, highlighting the importance of spatial heterogeneity in treatment response. Conclusions: This study demonstrates that informed lesion selection and tailored aggregation strategies significantly impact the predictive performance of radiomics-based models for progression and TTP prediction in PRRT-treated NET patients. These approaches can potentially enhance model accuracy and better capture tumor heterogeneity, supporting more personalized and practical PRRT implementation.

## Simple Summary

This study explored how aggregating radiomic features from multiple PET-identified lesions can predict disease progression and Time to Progression (TTP) in patients with Neuroendocrine Tumors (NETs) treated with [^177^Lu]Lu-DOTA-TATE. Using different lesion selection and aggregation strategies, we found that selecting the top lesions sorted by descending minimum Standard Uptake Value (SUV_min_) improved progression prediction, while aggregating all lesion features or using the top five by descending SUV_mean_ yielded more accurate TTP predictions. The best-performing models were Logistic Regression (LR) for progression and Random Survival Forest (RSF) for TTP, highlighting the value of spatial heterogeneity features. Overall, the findings show that strategic lesion selection and aggregation can enhance predictive accuracy and support more personalized PRRT treatment planning.

## 1. Introduction

Peptide Receptor Radionuclide Therapy (PRRT) has emerged as a pivotal treatment for patients with advanced Neuroendocrine Tumors (NETs), offering targeted cytotoxicity via radiolabeled somatostatin analogs [1]. The U.S. FDA-approved radiopharmaceutical [^177^Lu]Lu-DOTA-TATE specifically binds to somatostatin receptors (SST) expressing gastroenteropancreatic NETs and is used particularly in patients with progressive disease following conventional therapy [2]. Despite its clinical efficacy, the ability to accurately predict individual treatment outcomes remains limited.

Prior to the treatment, Positron Emission Tomography (PET) imaging using SSTR-targeted radiopharmaceuticals often uncovers a substantial tumor burden in patients. This typically includes not only the primary tumor, but also multiple metastatic lesions scattered throughout the body [3]. Each of these metastatic lesions can display unique inter- and intra-lesion biological characteristics and behaviors, such as differences in growth rate, receptor density, metabolic activity, and response to therapy [4]. These variations can significantly impact disease progression and treatment outcomes. Therefore, understanding and assessing the heterogeneity within and among these lesions is crucial for accurate prognosis and effective personalized treatment planning [5].

Radiomics provides a powerful, non-invasive means to quantitatively analyze imaging features from PET/CT scans, offering the potential to uncover prognostic and predictive biomarkers relevant to PRRT response [6]. Traditionally, analyses have centered on a single representative lesion, typically the largest or most metabolically active, potentially missing valuable clinical insights from other lesions [5]. However, determining the optimal approach to integrate radiomic data from multiple lesions within a single patient remains a challenge, particularly due to the variability in lesion count across individuals [3,4,7].

Several studies have shown that incorporating additional Regions of Interest (ROIs), such as other lesions [4,5,8], the peritumoral area [9,10], and even healthy organs [11] alongside the primary tumor can enhance outcome prediction. In this context, Captier et al. [4] introduced RadShap, a model- and modality-agnostic explanation tool for multiregional radiomic models, built on Shapley values [12]. RadShap assigns prediction contributions to individual image regions. Aggregative models using RadShap can outperform single-lesion models for both survival prediction and tumor subtype classification, highlighting the prognostic value of regions beyond the primary tumor. Wilk et al. [8] assessed integrating radiomic data from multiple lesions into survival models for lung cancer. Using PET and PET interpolated to CT resolution features from 115 patients, they tested two strategies: aggregating lesion features or combining model outputs. Survival models were evaluated with Monte Carlo cross-validation. Including all lesions significantly improved prediction (C-index from ~0.61 to ~0.63), showing added value beyond the primary tumor.

Salimi et al. [10] explored improving survival prediction using machine learning (ML) in 2926 head and neck cancer CT scans by including radiomic features from peritumoral tissue around the tumor. The results showed that adding peritumoral radiomics consistently improved prediction, increasing the C-index from 0.64 (tumor only) up to 0.68, highlighting the prognostic value of tissue surrounding the tumor. In another study, Salimi et al. [11] evaluated the added value of radiomic features extracted from healthy organs (referred to as Organomics, or non-tumor radiomics) in predicting survival for non-small cell lung cancer patients using PET/CT images and ML. Using data from 154 patients, features from tumors and 33 healthy organ regions were analyzed across various input combinations, feature selection methods, and ML models. The results showed that Organomics (especially from PET and CT) significantly contributed to model performance.

Despite these advances, there remains no standardized or validated approach for leveraging radiomic information from multiple lesions to predict progression and Time to Progression (TTP) in PRRT-treated patients. In this study, we hypothesize that metastatic lesions differ in their biological contribution to treatment outcome, and that ranking lesions according to imaging-derived markers of SSTR expression and tumor burden provides a meaningful way to identify those most informative for prediction. Guided by this rationale, we evaluated five lesion-ranking strategies: descending maximum Standard Uptake Value (SUV_max_), SUV_mean_, and lesion volume, as well as both descending and ascending SUV_min_. Uptake-based rankings reflect differences in receptor density and viable tumor activity, while lesion volume captures the prognostic impact of tumor burden. Including both high- and low-uptake SUV_min_ orderings enables assessment of lesions with minimal radiopharmaceutical accumulation, which may represent biologically distinct disease components.

This study systematically compares combinations of lesion prioritization and feature aggregation methods to determine how radiomic features from multiple lesions can be optimally utilized to predict disease progression and TTP following [^177^Lu]Lu-DOTA-TATE therapy. Our goal is to provide a robust framework that captures inter-tumor heterogeneity and improves model performance, ultimately supporting more accurate patient stratification for PRRT.

## 2. Materials and Methods

**Figure 1:**
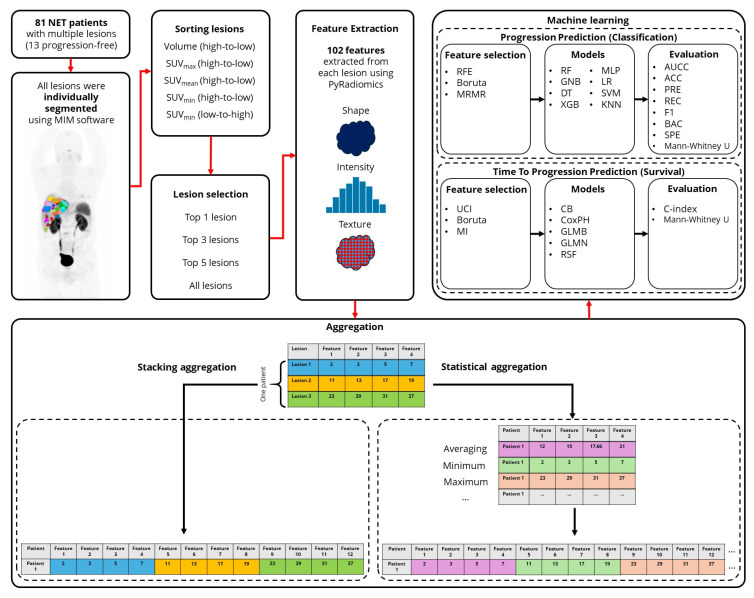
Workflow of the study design. (NET: Neuroendocrine Tumors, RFE: Recursive Feature Elimination, MRMR: Minimum Redundancy Maximum Relevance, UCI: Univariate C-Index, MI: Mutual Information, RF: Random Forest, GNB: Gaussian Naive Bayes, DT: Decision Tree, XGB: eXtreme Gradient Boosting, MLP: Multi-layer Perceptron, LR: Logistic Regression, SVM: Support Vector Machine, KNN: K-Nearest Neighbors, CB: CatBoost, CoxPH: Cox Proportional Hazards, GLMB: Generalized Linear Model Boosting, GLMN: Generalized Linear Model Net, RSF: Random Survival Forests, AUCC: Area Under the Characteristic Curve, ACC: Accuracy, PRE: Precision, REC: Recall, BAC: Balanced Accuracy, SPE: Specificity).

The overview of the current study is provided in Figure 1. In the following sections, we further elaborate our methods.

### 2.1. Data Collection

In this study, we retrospectively analyzed 81 NETs patients (13 progression-free) who underwent pre-treatment ^68^Ga-DOTA-TOC and ^68^Ga-DOTA-TATE PET/CT imaging (similar accuracy in detection of SSTR positivity [13]) followed by [^177^Lu]Lu-DOTA-TATE therapy. Detailed patients’ clinical data is provided in the study by Zamboni et al. [3]. In the current cohort of 81 patients, the median follow-up was 34 months (range 0.7–61.1 months). For the 24-month TTP analysis, 71 patients had evaluable data, 39 of which experienced progression, and 10 of them died.

The analysis was conducted in two phases: (1) classification of patients based on disease progression status (event vs. event-free), and (2) prediction of TTP to assess survival outcomes. Death was also considered as progression in both tasks.

### 2.2. Lesion Segmentation

All metastatic lesions were segmented using a standardized semi-automatic workflow (MIM Software Inc., Cleveland, OH, USA), which employs a gradient-based algorithm for tumor boundary detection. All lesions in our study were segmented using MIM gradient-based contouring (PETEdge and PETEdge+ tools) [14] which is recognized as a robust and reproducible method for PET tumor delineation [15,16,17,18], and proved to be more reproducible compared to manual segmentation [18]. An experienced investigator carefully verified every detected lesion, removed physiologic uptake, and segmented all lesions using PETEdge (for smaller lesions) and PETEdge+ (for larger, irregular shaped lesions) tools. To ensure consistency, all PET/CT studies were independently re-evaluated by a nuclear medicine physician with expertise in PRRT imaging for metastatic lesion calling. Although formal inter-observer variability metrics were not computed, this dual-review and consensus procedure minimized segmentation inconsistencies and ensured reliable lesion contours throughout the dataset.

### 2.3. Data Preparation

All images were converted from Bq to SUV. To manage the high-intensity injection site, SUV values were clipped based on the highest pixel value within all segmented lesions per patient. This allowed normalization to the 0–20 range based on global minimum and maximum across images without losing relevant signal, ensuring that lesion intensity information was preserved. Lesions were then ranked based on five criteria: descending SUV_max_, SUV_mean_, and lesion volume, as well as descending and ascending SUV_min_ (we refer to the ascending order as SUV_min__LH (low to high), while the descending order is simply denoted as SUV_min_). Notably, for SUV_min_, both descending and ascending orderings were considered to capture variations in lesion uptake characteristics, particularly those with the lowest radiopharmaceutical accumulation.

This sorting process yielded five distinct lesion ranking lists. From each list, the top 1, 3, and 5 lesions were selected for subsequent radiomic feature extraction and analysis, enabling evaluation of the impact of lesion selection strategy on predictive performance. The choice to analyze the top 1, 3, and 5 lesions for each ranking strategy was motivated by the need to balance biological interpretability with statistical feasibility. These subset sizes allowed us to evaluate whether adding a small number of highly ranked lesions provides incremental predictive value while avoiding the noise and overfitting risk associated with including larger numbers of lesions with potentially low clinical relevance. This approach also ensured methodological consistency across ranking strategies and aligns with prior multi-lesion radiomics studies that limit analysis to a small set of the most informative lesions [8].

### 2.4. Radiomic Features Extraction

One hundred and seven radiomic features, including shape, intensity, and texture from different families of shape (*n* = 14), First Order (FO, *n* = 18), Gray Level Co-occurrence Matrix (GLCM, *n* = 24), Gray Level Run Length Matrix (GLRLM, *n* = 16), Gray Level Size Zone Matrix (GLSZM, *n* = 16), Gray Level Dependence Matrix (GLDM, *n* = 14), and Neighboring Gray Tone Difference Matrix (NGTDM, *n* = 5) were extracted from each selected lesion using the PyRadiomics library (version 3.1.0) [19]. Table S1 in the Supplementary Material includes all the features. Feature extraction was performed using a bin width of 0.3125. Both images and segmentations were resampled to an isotropic voxel size of (2, 2, 2) mm^3^, using B-spline interpolation for images and nearest-neighbor interpolation for segmentations. A masked voxel kernel with a radius of 3 was applied to restrict calculations to within the regions of interest (ROIs).

### 2.5. Lesion Aggregation

To aggregate lesion-level information, two approaches were employed: stacking and statistical aggregation. In the stacking method, feature values from the top 3 and top 5 lesions (including SUVs, volume, and radiomics) were concatenated horizontally to form a combined feature vector. In contrast, statistical aggregation summarized the features across the selected lesions using the key descriptive statistics of minimum, maximum, mean, median, variance, skewness, kurtosis, and coefficient of variation in each feature along the selected lesions and the lesions concatenated horizontally. As expected, aggregation was not applicable for the top 1 lesion dataset, where only features from an individual lesion were available. Additionally, to evaluate the potential benefit of incorporating information from all lesions, a separate dataset was created by statistically aggregating features across all segmented lesions per patient. This allowed assessment of whether comprehensive lesion inclusion enhances model performance.

### 2.6. Progression Prediction

The dataset was normalized and subjected to a nested cross-validation framework comprising 4 outer loops and 3 inner loops. The Z-score normalization was performed on train dataset and then the mean and standard deviation transformed to test in each outer fold. Class imbalance was addressed using the Synthetic Minority Oversampling Technique (SMOTE) [20] on the train dataset in the inner fold during training. To reduce redundancy, highly correlated features (Spearman correlation > 0.80 lead to the best results) were removed. Multiple correlation thresholds were evaluated during preliminary experiments, and the cutoff of 0.80 for progression prediction provided the best balance between removing redundant features and preserving informative variability across patients. Feature selection was performed iteratively to identify the 15 most predictive features (lead to the best results) using three algorithms: Boruta [21] and Recursive Feature Elimination (RFE) [22], both with a Random Forest (RF) [23] core, and Minimum Redundancy Maximum Relevance (MRMR) [24]. Limiting the final feature subset to 15 features was based on systematic testing across several feature-selection methods, where this number consistently produced the most stable performance across nested cross-validation and bootstrapping while preventing overfitting.

In each fold, the selected features were input into eight ML classifiers: RF, Gaussian Naive Bayes (GNB) [25], Decision Tree (DT) [26], eXtreme Gradient Boosting (XGB) [27], Multi-layer Perceptron (MLP) [28], Logistic Regression (LR) [29], Support Vector Machine (SVM) [30], and K-Nearest Neighbors (KNN) [31]. Hyperparameter tuning was conducted via grid search and 3 inner folds cross-validation. Model performance was evaluated after 1000 bootstrapping on each test fold using several metrics: Area Under the Characteristic Curve (AUCC), accuracy (ACC), precision, recall, F1-score, balanced accuracy (BAC), and specificity. To compare model performances, the Mann–Whitney U test was applied, and *p*-values were corrected for multiple comparisons using the False Discovery Rate (FDR) method by Benjamini–Hochberg [32].

### 2.7. Time to Progression

After the normalization, the highly correlated features (Spearman correlation > 0.95 lead to the best results) were removed and the maximum selected features in each fold was 8 (this led to the best results). The correlation threshold for TTP prediction was selected because survival modeling is more sensitive to redundancy and sample-size constraints. Preliminary evaluation of several thresholds showed that 0.95 produced the highest stability and reproducibility. Likewise, capping the selected features at 8 was determined empirically, as this level maximized predictive performance across feature-selection techniques while maintaining an appropriate ratio of predictors to events for survival analysis.

The same nested cross-validation framework was applied for TTP prediction. Feature selection methods included Boruta, Univariate C-Index (UCI) [33], and Mutual Information (MI) [34]. These features were then used to train five survival models: CatBoost (CB) [35], Cox Proportional Hazards (CoxPH) [36], Generalized Linear Model Boosting (GLMB) [37], Generalized Linear Model Net (GLMN) [38], and Random Survival Forests (RSF) [39]. Hyperparameter tuning was conducted via grid search and model performance was assessed using 1000 bootstrapping on the test folds using C-index. Comparisons between models were conducted using the Mann–Whitney U test, with Benjamini–Hochberg FDR correction used to adjust for multiple testing.

## 3. Results

Table 1 shows the location of selected lesions based on the different sorting methods including SUV_min__LH, SUV_min_, SUV_max_, SUV_mean_, and volume. Across all sorting strategies, liver metastases represented the largest proportion of selected lesions, followed by bone and lymph nodes. Methods prioritizing higher uptake or larger lesions (SUV_max_, SUV_mean_, volume) consistently selected a greater number of liver lesions, whereas SUV_min_-based ranking yielded a more diverse distribution that captured a higher proportion of bone, peritoneal, and soft-tissue lesions. This pattern persisted when expanding the selection from the top one to the top three and top five lesions, indicating that SUV_min_-based ranking tends to highlight biologically heterogeneous lesions across multiple anatomical sites rather than focusing predominantly on liver-related lesions. The overall distribution underscores the variability in lesion selection driven by different ranking strategies and supports the premise that multi-lesion analysis captures a broader spectrum of disease heterogeneity.

The results of this study are presented in overall and individual schemes. The overall appraisal of the results provides a general idea of how the ML models performed being trained on different datasets, aggregation strategies, and sorting methods by considering the evaluation metrics averaged over all combinations of ML algorithms and feature selections. However, the individual assessment attempts to introduce the best performing models in each dataset along with the most important selected features.

### 3.1. Progression Prediction

#### 3.1.1. Overall Appraisal

Table 2 shows the evaluation metrics averaged on all combinations of ML algorithms and feature selections for the top three sorting and aggregation strategies used to classify disease progression status (event vs. event-free) in each dataset. Comparing the average performance of the models, it is evident that sorting lesions based on the SUV_min_ and volume is a better lesion selection strategy than other sorting approaches in all the datasets. For instance, using radiomic features extracted from the top one lesion sorted based on SUV_min-_enabled ML models show the highest overall performance.

**Figure 2:**
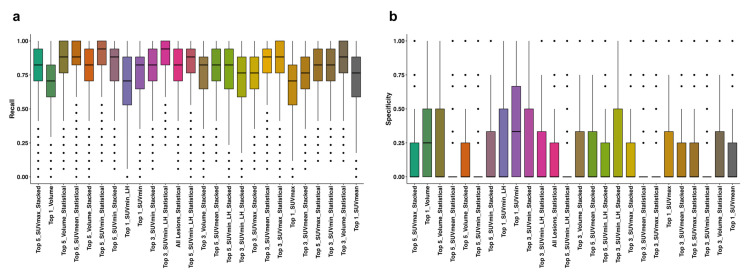
Distribution of recall (**a**) and specificity (**b**) values of all the models in each dataset, aggregation, and sorting strategies. The horizontal line within each box indicates the median, while the box edges represent the interquartile range (25th to 75th percentiles). Whiskers extend to data points within 1.5× the interquartile range, and individual dots represent outliers. SUV: Standardized Uptake Value, LH: Low-to-High.

Although there are performance differences between lesion selection strategies, missing a progressing patient has direct implications for treatment decisions and follow-up planning. Models trained on lesions ranked by SUV_min_ consistently achieved higher recall, indicating better sensitivity to early biological changes associated with progression. The observed gap between recall and specificity reflects the limited number of progression-free patients, which creates a substantial class imbalance. To mitigate this, SMOTE was applied during training; however, some instability is expected when the minority class is small. Distribution of the average recall and specificity values of the models are shown in Figure 2a,b.

**Figure 3:**
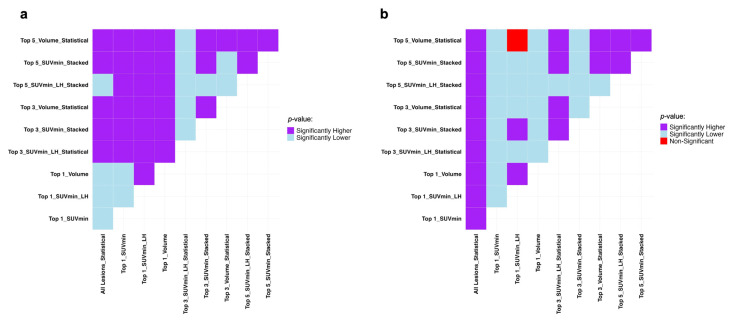
Mann–Whitney U test results comparing the average machine performance trained on all the datasets and sorting approaches used in progression event prediction presented in Table 2 regarding (**a**) recall and (**b**) specificity. It compares row (Model A) vs. column (Model B): purple = A significantly better, light blue = A significantly worse, red = No difference. SUV: Standardized Uptake Value, LH: Low-to-High.

The Mann–Whitney U test results presented in Figure 3 show if the difference in datasets and sorting in Table 2 are statistically significant in terms of recall and specificity. Generally, the single top lesion sorted based on SUV_min_ gives superior recall and specificity. A more inclusive picture of the comparison between the average performance of all the models in each lesion selection and sorting approaches is provided in Figure S1 in the Supplementary Material.

#### 3.1.2. Individual Appraisal

Table 3 shows evaluation metrics of the top three ML models with the highest performance used in classifying patients based on disease progression status (event vs. event-free) in each dataset along with the most important selected features. The universal-best model turned out to be RFE_LR trained on the radiomic features of a single lesion sorted based on SUV_min_.

SUV_min_-sorted lesions could also result in higher predictive power in the top five dataset. This is while sorting based on lesion volume could give more predictive models in the top three dataset. According to Table 3, lesion selection proved to be effective in enhancing machine performance. Also, stacking radiomic features appeared to be a better way of feature aggregation in both top three and top five datasets.

**Figure 4:**
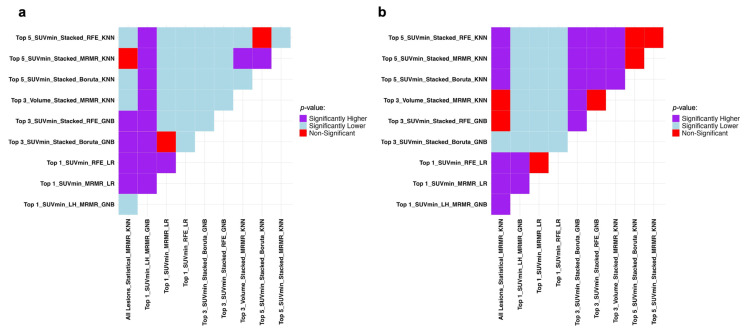
Mann–Whitney U test results comparing the models with the highest performance metrics in progression prediction presented in Table 3 regarding (**a**) recall and (**b**) specificity. It compares row (Model A) vs. column (Model B): purple = A significantly better, light blue = A significantly worse, red = No difference. SUV: Standardized Uptake Value, RFE: Recursive Feature Elimination, LR: Logistic Regression, MRMR: Minimum Redundancy Maximum Relevance, GNB: Gaussian Naive Bayes, KNN: K-Nearest Neighbors.

The Mann–Whitney U test results in Figure 4 prove the superiority of the Top_1_SUV_min__RFE_LR combination compared to other combinations presented in Table 3 regarding recall and specificity.

Turning to the most important features, it is immediately obvious that at least one feature from GLSZM and shape families were among the highly predictive features almost every time regardless of the dataset or feature selection method. Moreover, in the stacked-aggerated top three and top five datasets, a greater number of features were selected from the topper lesions. This indicates that lesion importance is directly related to its rank.

### 3.2. Time to Progression

#### 3.2.1. Overall Appraisal

Table 4 shows C-indices averaged on all combinations of ML algorithms and feature selections for the top three sorting and aggregation strategies used for TTP analysis. The average performance of the models shows that statistical aggregation of features extracted from all lesions leads to the highest overall performance. Sorting the top five lesions based on the SUV_mean_ can also yield the same results. This highlights the fact that including all lesions does not necessarily improve the model performance in SSTR PET.

**Figure 5:**
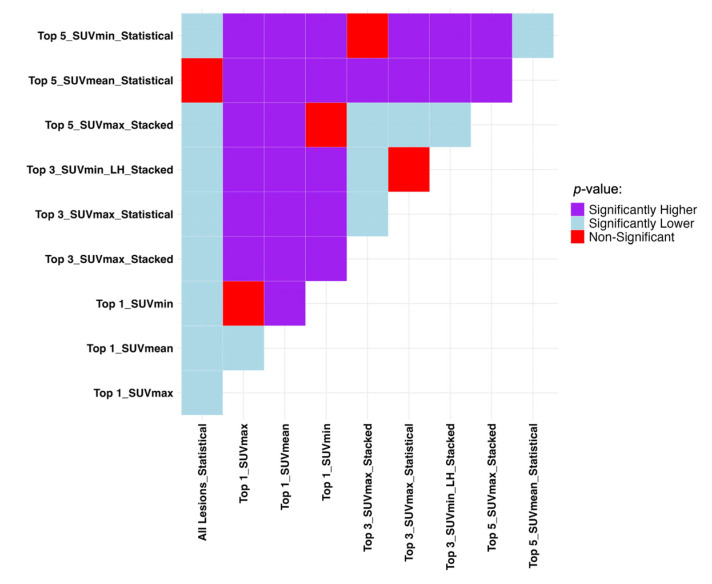
Mann–Whitney U test results comparing the top 3 sorting and aggregation approaches in each dataset with the highest average performance in TTP analysis regarding C-index. It compares row (Model A) vs. column (Model B): purple = A significantly better, light blue = A significantly worse, red = No difference. SUV: Standardized Uptake Value, LH: Low-to-High.

The Mann–Whitney U test results presented in Figure 5 show if the difference in datasets and sorting in Table 4 are statistically significant. Due to the statistically insignificant difference between overall machine performance between when it is trained on all features and when only the top five SUV_mean_-sorted lesions are selected, the top five dataset can be used instead. A more inclusive picture of the comparison between the average performance of all the models on each lesion selection and sorting approaches is provided in Figure S2 in the Supplementary Material.

#### 3.2.2. Individual Appraisal

Table 5 shows the C-indices of the top three ML models with the highest performance in TTP analysis along with the most important selected features. The universal-best model appeared to be UCI_RSF trained on the statistically aggregated features of the top five lesions sorted based on SUV_mean_.

**Figure 6:**
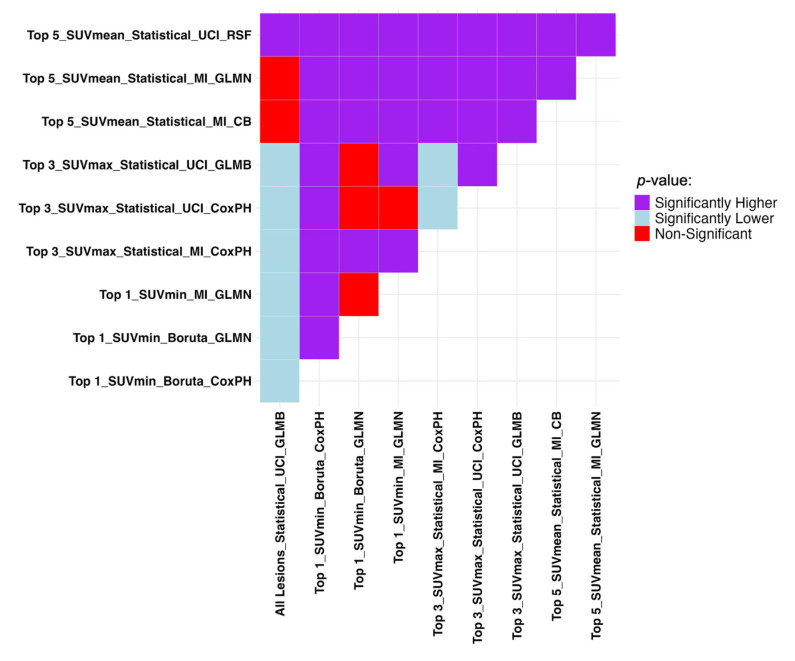
Mann–Whitney U test results comparing the top 3 ML models and features in each dataset with the highest performance in TTP analysis regarding C-indices. It compares row (Model A) vs. column (Model B): purple = A significantly better, light blue = A significantly worse, red = No difference. SUV: Standardized Uptake Value, LH: Low-to-High. GLMN: Generalized Linear Model Net, MI: Mutual Information, CoxPH: Cox Proportional Hazards, UCI: Univariate C-Index, GLMB: Generalized Linear Model Boosting, RSF: Random Survival Forests, CB: CatBoost.

This highlights the fact that higher performance can also be achieved in TTP studies by including a smaller number of lesions. SUV_min_ and SUV_max_ can also lead to acceptable results depending on the number of lesions selected. According to Table 5, enhancing machine performance is possible by lesion selection. In contrast to progression prediction analysis, models performed better in TTP when fed by statistically aggregated features. Figure 6 shows Mann–Whitney U test results comparing the models presented in Table 5.

The important selected features in Table 5 highlight the importance of GLSZM and GLDM families. Measures of variability such as kurtosis, skewness, and standard deviation turned out to be better ways of aggregation as most of the important features were such aggregations of original radiomic features among the selected lesions.

## 4. Discussion

This study presented a comprehensive evaluation of lesion selection and feature aggregation strategies for radiomics-based prediction of progression and TTP in patients with NETs undergoing [^177^Lu]Lu-DOTA-TATE PRRT. While prior work in PET radiomics has largely focused on a single lesion per patient, often selected based on size or metabolic activity [3], our results demonstrate that the method of lesion selection and aggregation significantly impacts model performance, which is also in a good agreement with other studies [3,4,5,8]. This finding emphasizes the need for carefully designed frameworks to better capture the complexity of tumor heterogeneity in NETs.

Traditionally, radiomics studies have relied on features extracted from a single representative lesion, typically the largest or the one with the highest SUV [3]. However, this approach may miss critical biological heterogeneity present across the full disease burden. Our results show that lesion selection based on SUV_min_ consistently yielded better performance in progression prediction than SUV_max_ or volume. This suggests that lesions with low uptake which potentially represent dedifferentiated or less receptor-expressing clones may carry significant prognostic value. Such lesions might be more resistant to PRRT, contributing disproportionately to disease progression despite their smaller size or lower visual prominence [40]. Including these SUV_min_ lesions enhances model sensitivity to biologically aggressive disease. Moreover, methods based on higher uptake or larger size mainly picked liver lesions, while SUV_min_ (Table 1) captured more bone, peritoneal, and soft-tissue sites. This trend held from the top one through top five selections, showing that SUV_min_ favors more biologically heterogeneous lesions rather than focusing predominantly on liver-related lesions.

Furthermore, the results of this study indicate that using multi-lesion radiomic features, rather than focusing on a single lesion, can yield improved outcomes in TTP prediction, as demonstrated in Table 4 and Table 5. Another finding of our study was the association between SUV_mean_ and TTP. Previous studies have shown that higher SUV_mean_ values predict better response to PRRT in the NETs [41,42], as well as higher lesion absorbed dose [43]. In our study, again, according to Table 4 and Table 5, both in overall and individual analysis of models, it is obvious that SUV_mean_-based sorting led to the superior results.

This supports recent arguments in the literature that highlight the limitations of “largest-lesion” heuristics and advocate for data-driven lesion prioritization strategies that reflect clinically relevant biology [4]. Our findings reinforce the need to revise radiomics workflows to move beyond a “one-lesion-fits-all” approach and adopt more nuanced lesion ranking criteria.

Another key contribution of this work is the comparison of stacking versus statistical aggregation techniques for multi-lesion data integration. For progression classification, stacking features across the top-ranked lesions yielded superior results in some scenarios, particularly in the top three and top five lesion datasets. This approach retains lesion-specific signatures that might otherwise be lost in statistical summaries. However, for TTP prediction, statistical aggregation consistently outperformed stacking. Models trained on statistical descriptors (e.g., mean, skewness, kurtosis) of radiomic features across lesions achieved higher C-indices, indicating better survival prediction.

These findings suggest that task-specific aggregation strategies are necessary: detailed lesion-level granularity and shape may benefit classification tasks with discrete outcomes, while statistical summaries may better capture global tumor dynamics relevant to longitudinal endpoints like progression time. Notably, aggregating features from only the top five lesions, ranked by SUV_mean_, produced comparable to using all lesions, which is a result with important implications for clinical and computational feasibility.

Consistently across both tasks, features from GLSZM category were among the most predictive. GLDM family was also important in TTP analysis. These texture matrices capture patterns of intra-tumoral heterogeneity and spatial distribution of uptake intensities. Specifically, GLSZM reflects the size and distribution of zones with similar uptake intensity, while GLDM quantifies the degree of gray-level differences between neighboring voxels [44]. These features may indicate how uniform or heterogeneous the tumor is in terms of tracer uptake, which could be associated with biological behavior such as aggressiveness, vascularity, cellular density, and somatostatin receptor density in the case of SSRT PET. The selection of radiomic features such as GLSZM_SZNUN and GLDM_SDE, which reflect non-uniformity and dependency strength, respectively, highlights the role of spatial disorder in predicting PRRT outcomes. This complements biological insights from prior research that intra-patient and intra-tumoral heterogeneity, as reflected in PET imaging, correlates with more aggressive disease and poor therapeutic response and prognosis in various cancers [2,4]. While texture features such as GLSZM and GLDM showed strong predictive value, their biological interpretation should be viewed cautiously. These metrics likely capture aspects of intra-tumoral heterogeneity, but their exact correspondence to histopathological or molecular characteristics remains an area of ongoing research, and definitive biological links cannot be assumed.

Moreover, several highly ranked features involved higher-order statistics derived by applying statistical measures like kurtosis, skewness, and standard deviation to the radiomic values across lesions. These aggregation metrics reflect the variability of radiomic expression within and across lesions that may encode biologically meaningful patterns related to receptor expression, dedifferentiation, or treatment resistance. For example, high skewness might suggest that some lesions behave very differently from others, high kurtosis could indicate outlier lesions with extreme characteristics, and standard deviation is able to capture the overall heterogeneity. From a clinical standpoint, such variability may reflect heterogeneous biology, variable treatment response, or the presence of aggressive subclones, thus offering interpretable indices that can support decision-making in managing NETs.

Several prior studies have demonstrated that incorporating radiomic features from regions beyond the primary lesion can improve predictive performance. For example, Wilk et al. [8] showed that including all lesions significantly enhanced survival model performance in lung cancer patients. Our findings are consistent with these results and further extend them by introducing and systematically evaluating lesion ranking and aggregation methods tailored for PRRT.

In contrast to studies incorporating peritumoral [9,10] or non-tumor/healthy-organ radiomics [11,45], our study focused on lesion-based modeling. However, these complementary approaches underscore the growing recognition that prognostically relevant information may exist outside the tumor itself.

The study provides practical recommendations for radiomics implementation in the context of PRRT. First, our findings suggest that a small, systematically selected subset of lesions can offer similar or even better predictive power than all-lesion approaches, enabling more efficient workflows. This is particularly valuable in NETs, where metastatic burden is often extensive, and manual lesion segmentation is labor-intensive. Second, lesion selection based on SUV_min_ (progression prediction task) or SUV_mean_ (TTP prediction task), rather than SUV_max_ or volume alone, appears to better stratify risk and capture clinically relevant disease biology. Finally, statistical aggregation of features, particularly for TTP modeling, may offer a scalable and interpretable alternative to stacking, reducing model complexity and potential overfitting, especially in modestly sized datasets.

These insights are directly aligned with the broader aim of radiomics: to derive quantitative biomarkers from imaging that reflect tumor biology, support treatment decisions, and improve patient stratification in personalized oncology [4].

To evaluate the transparency and reproducibility of our study, we applied the METRICS checklist [46] and achieved a score of 68.2%, placing it in the “Good” quality category (Figures S3 and S4). This indicates strong performance in model validation, feature robustness, and clinical relevance, while also identifying areas for improvement in reproducibility and standardization.

This study has several limitations. Although nested cross-validation with bootstrapping was employed to mitigate bias, external validation on independent cohorts is essential. The number of patients who remained progression-free was relatively small (n = 13), potentially affecting model calibration and sensitivity to class imbalance. Furthermore, while this study focused exclusively on imaging features, integrating clinical, molecular, or histopathologic data could enhance predictive accuracy. In addition, incorporating Explainable AI (XAI) frameworks could aid in translating complex radiomic models into clinically actionable tools.

Future studies may explore automated lesion detection, reduce manual segmentation efforts, and enhance reproducibility. Moreover, longitudinal imaging and temporal radiomics could further refine TTP predictions by capturing dynamic treatment response. Another important consideration is model generalizability. Radiomics can be sensitive to variations in scanners, reconstruction protocols, segmentation workflows, and institutional acquisition differences. Although our nested cross-validation reduces overfitting within this dataset, confirming robustness would require harmonized multicenter cohorts, standardized imaging, and reconstruction protocols, and external validation on datasets acquired under different clinical conditions. Such steps are essential before these models can be translated into broadly applicable clinical tools [47,48]. Future research could also explore combining lesion extension and feature aggregation to evaluate its performance on progression and TTP prediction. Finally, our lesion selection and aggregation methodology may be extended to other radiopharmaceutical therapies and tumor types where heterogeneous tumor burden is prevalent.

## 5. Conclusions

This study demonstrated that lesion selection and feature aggregation strategies significantly influence the predictive performance of radiomics-based models for disease progression and TTP in NET patients undergoing PRRT. Selecting a single lesion based on SUV_min_ optimizes classification performance, while using the top five lesions ranked by SUV_mean_ with statistical aggregation enhances survival prediction. Features from GLSZM and GLDM families were consistently important, underscoring the value of spatial heterogeneity in risk stratification. These findings support the use of targeted, data-driven lesion selection, and task-specific aggregation approaches to improve efficiency, accuracy, and clinical applicability of radiomics in personalized therapy.

## Figures and Tables

**Table 1:** Anatomical distribution of lesions selected according to each ranking strategy (SUV_min__LH, SUV_min_, SUV_max_, SUV_mean_, and volume), illustrating how lesion location varies across the different sorting methods.

Sorting Method	Liver	Bone	Lymph Node	Peritoneum	Soft Tissue
Top 1 SUV_min__LH	35	23	11	8	4
Top 1 SUV_min_	44	8	13	2	14
Top 1 SUV_max_	47	5	15	3	11
Top 1 SUV_mean_	49	4	14	1	13
Top 1 Volume	50	6	12	3	10
Top 3 SUV_min__LH	101	64	34	23	20
Top 3 SUV_min_	141	18	42	8	33
Top 3 SUV_max_	132	17	60	7	26
Top 3 SUV_mean_	140	14	49	9	30
Top 3 Volume	151	13	40	10	28
Top 5 SUV_min__LH	175	105	45	41	34
Top 5 SUV_min_	240	29	70	14	47
Top 5 SUV_max_	221	29	88	14	48
Top 5 SUV_mean_	229	27	82	14	48
Top 5 Volume	259	23	64	18	36
Overall	2627	1143	525	401	307

SUV: Standardized Uptake Value, LH: Low-to-High.

**Table 2:** The top 3 sorting and aggregation approaches in each dataset with the highest average performance metrics in progression prediction.

Dataset	Sorting	Aggregation	AUCC ± SD	ACC ± SD	BAC ± SD	REC ± SD	SPE ± SD	PRE ± SD	F1-Score ± SD
Top 1	**SUV_min_**	-	**0.61 ± 0.23**	**0.70 ± 0.16**	**0.58 ± 0.19**	**0.76 ± 0.20**	**0.41 ± 0.39**	**0.87 ± 0.10**	**0.79 ± 0.14**
	Volume	-	0.43 ± 0.23	0.64 ± 0.15	0.49 ± 0.16	0.70 ± 0.19	0.28 ± 0.32	0.84 ± 0.08	0.75 ± 0.14
	SUV_min__LH	-	0.43 ± 0.22	0.61 ± 0.20	0.46 ± 0.17	0.67 ± 0.25	0.25 ± 0.33	0.81 ± 0.14	0.71 ± 0.21
Top 3	SUV_min_	Stacked	0.59 ± 0.19	0.72 ± 0.13	0.53 ± 0.15	0.81 ± 0.17	0.25 ± 0.32	0.85 ± 0.06	0.82 ± 0.10
	SUV_min__LH	Statistical	0.53 ± 0.22	0.77 ± 0.10	0.52 ± 0.13	0.88 ± 0.12	0.16 ± 0.25	0.85 ± 0.05	0.86 ± 0.07
	Volume	Statistical	0.53 ± 0.26	0.75 ± 0.12	0.53 ± 0.16	0.86 ± 0.13	0.20 ± 0.29	0.85 ± 0.06	0.85 ± 0.09
Top 5	Volume	Statistical	0.58 ± 0.23	0.75 ± 0.13	0.55 ± 0.16	0.85 ± 0.16	0.25 ± 0.32	0.86 ± 0.06	0.85 ± 0.10
	SUV_min_	Stacked	0.60 ± 0.21	0.73 ± 0.13	0.53 ± 0.16	0.82 ± 0.17	0.23 ± 0.34	0.85 ± 0.07	0.83 ± 0.11
	SUV_min__LH	Statistical	0.46 ± 0.20	0.71 ± 0.12	0.47 ± 0.12	0.83 ± 0.15	0.12 ± 0.23	0.83 ± 0.05	0.82 ± 0.09
All Lesions	-	Statistical	0.47 ± 0.22	0.69 ± 0.13	0.47 ± 0.14	0.80 ± 0.16	0.15 ± 0.27	0.83 ± 0.06	0.80 ± 0.11

AUCC: Area Under the Characteristic Curve, ACC: Accuracy, BAC: Balanced Accuracy, REC: Recall, SPE: Specificity, PRE: Precision, SD: Standard Deviation, SUV: Standardized Uptake Value, LH: Low-to-High.

**Table 3:** The top 3 ML models and features in each dataset with the highest performance metrics in progression prediction.

Set	Sorting	Aggregation	FS	Important Features	Classifier	AUCC ± SD	ACC ± SD	BAC ± SD	REC ± SD	SPE ± SD	PRE ± SD	F1-Score ± SD
Top 1	**SUV_min_**	-	**RFE**	**GLSZM_SZNUN, GLCM_IV, SUVmax**	**LR**	**0.71 ± 0.22**	**0.75 ± 0.11**	**0.76 ± 0.12**	**0.75 ± 0.16**	**0.77 ± 0.32**	**0.96 ± 0.06**	**0.83 ± 0.09**
			MRMR	GLSZM_SZNUN, GLSZM_SALGLE, Shape_Sphericity	LR	0.69 ± 0.22	0.73 ± 0.13	0.74 ± 0.12	0.72 ± 0.18	0.77 ± 0.32	0.95 ± 0.06	0.80 ± 0.12
					GNB	0.44 ± 0.22	0.73 ± 0.18	0.48 ± 0.19	0.25 ± 0.20	0.71 ± 0.29	0.74 ± 0.33	0.35 ± 0.24
Top 3	Volume	Stacked	MRMR	Shape_Sphericity_3, Shape_Elongation_1, SUVmin_1	KNN	0.58 ± 0.18	0.56 ± 0.11	0.56 ± 0.17	0.56 ± 0.12	0.56 ± 0.32	0.87 ± 0.10	0.67 ± 0.10
			RFE	GLSZM_SAE_2, Shape_Elongation_1, SUVmin_1	GNB	0.57 ± 0.17	0.65 ± 0.12	0.61 ± 0.15	0.68 ± 0.18	0.54 ± 0.41	0.90 ± 0.08	0.75 ± 0.12
			Boruta	GLDM_DV_1, GLSZM_SAE_2, Shape_MAL_1	GNB	0.59 ± 0.22	0.68 ± 0.12	0.60 ± 0.17	0.72 ± 0.17	0.48 ± 0.42	0.89 ± 0.09	0.78 ± 0.10
Top 5	SUV_min_	Stacked	Boruta	Shape_Elongation_2, GLSZM_SALGLE_3, GLCM_IMC1_3	KNN	0.56 ± 0.19	0.54 ± 0.13	0.58 ± 0.15	0.53 ± 0.16	0.62 ± 0.28	0.88 ± 0.09	0.65 ± 0.13
			RFE	Shape_Elongation_5, GLSZM_SZNUN_1, GLCM_IMC1_3	KNN	0.57 ± 0.24	0.53 ± 0.13	0.56 ± 0.21	0.53 ± 0.14	0.59 ± 0.41	0.87 ± 0.13	0.64 ± 0.12
			MRMR	GLCM_IMC1_3, Shape_Elongation_2, GLSZM_SZNUN_1	KNN	0.62 ± 0.24	0.58 ± 0.12	0.58 ± 0.22	0.57 ± 0.13	0.58 ± 0.45	0.90 ± 0.11	0.69 ± 0.11
All Lesions	-	Statistical	MRMR	GLDM_DV_max, FO_Kurtosis_median, Shape_Elongation_kurtosis	KNN	0.53 ± 0.21	0.57 ± 0.13	0.56 ± 0.17	0.57 ± 0.18	0.54 ± 0.40	0.89 ± 0.10	0.67 ± 0.15

FS: Feature Selection, AUCC: Area Under the Characteristic Curve, ACC: Accuracy, BAC: Balanced Accuracy, REC: Recall, SPE: Specificity, PRE: Precision, SD: Standard Deviation, SUV: Standardized Uptake Value, RFE: Recursive Feature Elimination, LR: Logistic Regression, MRMR: Minimum Redundancy Maximum Relevance, GNB: Gaussian Naive Bayes, KNN: K-Nearest Neighbors.

**Table 4:** The top 3 sorting and aggregation approaches in each dataset with the highest average performance in TTP analysis.

Dataset	Sorting	Aggregation	C-Index ± SD
Top 1	SUV_min_	-	0.61 ± 0.03
	SUV_max_	-	0.60 ± 0.01
	SUV_mean_	-	0.59 ± 0.02
Top 3	SUV_max_	Stacked	0.61 ± 0.01
	SUV_max_	Statistical	0.61 ± 0.03
	SUV_min__LH	Stacked	0.60 ± 0.01
Top 5	**SUV_mean_**	**Statistical**	**0.65 ± 0.03**
	SUV_min_	Statistical	0.62 ± 0.03
	SUV_max_	Stacked	0.60 ± 0.02
All Lesions	-	**Statistical**	**0.65 ± 0.02**

SD: Standard Deviation, SUV: Standardized Uptake Value, LH: Low-to-High.

**Table 5:** The top 3 ML models and features in each dataset with the highest performance in TTP analysis.

Dataset	Sorting	Aggregation	FS	Top Features	Algorithm	C-Index ± SD
Top 1	SUV_min_	-	MI	GLSZM_ZE, GLSZM_SZNU, FO_Skewness	GLMN	0.64 ± 0.09
	SUV_min_	-	Boruta	GLDM_SDE, GLDM_SDHGLE, FO_Mean	GLMN	0.64 ± 0.08
	SUV_min_	-			CoxPH	0.63 ± 0.09
Top 3	SUV_max_	Statistical	MI	GLDM_GLNU_kurtosis, GLSZM_LAE_kurtosis, GLSZM_LAHGLE_kurtosis	CoxPH	0.66 ± 0.09
	SUV_max_	Statistical	UCI	GLCM_JointEntropy_std, GLCM_SS_cov, GLCM_SE_std	CoxPH	0.64 ± 0.09
	SUV_max_	Statistical			GLMB	0.64 ± 0.09
Top 5	**SUV_mean_**	**Statistical**	**UCI**	**GLSZM_ZP_skew, GLDM_SDE_skew, GLCM_DV_cov,**	**RSF**	**0.68 ± 0.09**
	SUV_mean_	Statistical	MI	GLSZM_ZP_skew, GLDM_SDE_skew, GLSZM_SZNU_min	CB	0.67 ± 0.09
	SUV_mean_	Statistical			GLMN	0.67 ± 0.10
All Lesions	-	Statistical	UCI	SUVmin_kurtosis, FO_Minimum_kurtosis, FO_Skewness_cov	GLMB	0.67 ± 0.12

SUV: Standardized Uptake Value, GLMN: Generalized Linear Model Net, MI: Mutual Information, CoxPH: Cox Proportional Hazards, UCI: Univariate C-Index, GLMB: Generalized Linear Model Boosting, RSF: Random Survival Forests, CB: CatBoost.

## Data Availability

Data is available upon reasonable requests.

## Supplementary Materials

The following supporting information can be downloaded at https://www.mdpi.com/article/10.3390/cancers17233887/s1, Table S1. Radiomic features adopted.; Figure S1. Mann-Whitney U test results comparing the average machine performance trained on all the datasets and sorting approaches used in progression event prediction regarding (a) recall and (b) specificity. It compares row (Model A) vs. column (Model B): purple = A significantly better, light blue = A significantly worse, red = No difference. SUV: Standardized Uptake Value, LH: Low-to-High.; Figure S2. Mann-Whitney U test results comparing the average machine performance trained on all the datasets and sorting approaches used in time to progression (TTP) prediction regarding C-index. It compares row (Model A) vs. column (Model B): purple = A significantly better, light blue = A significantly worse, red = No difference. SUV: Standardized Uptake Value, LH: Low-to-High.; Figure S3. First page of METRICS checklist.; Figure S4. Second page of METRICS checklist.

## Abbreviations

The following abbreviations are used in this manuscript:

PRRT	Peptide Receptor Radionuclide Therapy
NETs	Neuroendocrine Tumors
FDA	Food and Drug Administration
[^177^Lu]Lu-DOTA-TATE	Lutetium-177 DOTA-TATE
SSTR	Somatostatin Receptor
PET	Positron Emission Tomography
CT	Computed Tomography
ROI/ROIs	Region(s) of Interest
RadShap	Radiomics Shapley (model explanation tool)
ML	Machine Learning
TTP	Time to Progression
Bq	Becquerel
SUV/SUVs	Standardized Uptake Value(s)
SUVmin	Minimum Standardized Uptake Value
SUVmean	Mean Standardized Uptake Value
SUVmax	Maximum Standardized Uptake Value
GLCM	Gray Level Co-occurrence Matrix
GLRLM	Gray Level Run Length Matrix
GLSZM	Gray Level Size Zone Matrix
GLDM	Gray Level Dependence Matrix
NGTDM	Neighboring Gray Tone Difference Matrix
FO	First Order
SMOTE	Synthetic Minority Oversampling Technique
RFE	Recursive Feature Elimination
MRMR	Minimum Redundancy Maximum Relevance
RF	Random Forest
GNB	Gaussian Naive Bayes
DT	Decision Tree
XGB	eXtreme Gradient Boosting
MLP	Multi-Layer Perceptron
LR	Logistic Regression
SVM	Support Vector Machine
KNN	K-Nearest Neighbors
AUC/AUCC	Area Under the Curve/Area Under the Characteristic Curve
ACC	Accuracy
BAC	Balanced Accuracy
FDR	False Discovery Rate
UCI	Univariate C-Index
MI	Mutual Information
CB	CatBoost
CoxPH	Cox Proportional Hazards
GLMB	Generalized Linear Model Boosting
GLMN	Generalized Linear Model Net
RSF	Random Survival Forest
AI	Artificial Intelligence
XAI	Explainable Artificial Intelligence
