# Supplementary material for: Enhancing PRRT Outcome Prediction in Neuroendocrine Tumors: Aggregated Multi-Lesion PET Radiomics Incorporating Inter-Tumor Heterogeneity
Source: Cancers (Basel). 2025 Dec 4;17(23):3887. doi: 10.3390/cancers17233887 (PMC12690984; doi:10.3390/cancers17233887)
Supplement: Supplementary file 1 [file cancers-17-03887-s001.zip › cancers-3992693-supplementary.pdf]

Table S1. Radiomic features adopted.

| Family | # | Name | Family | # | Name |
|---|---|---|---|---|---|
| Gray level run length matrix (GLRLM) | 1 | Gray Level Non-Uniformity (GLNU) | Gray Level Size Zone Matrix (GLSZM) | 1 | Gray Level Non-Uniformity (GLNU) |
| | 2 | Gray Level Non-Uniformity Normalized (GLNUN) | | 2 | Gray Level Non-Uniformity Normalized (GLNUN) |
| | 3 | Gray Level Variance (GLV) | | 3 | Gray Level Variance (GLV) |
| | 4 | High Gray Level Run Emphasis (HGLRE) | | 4 | High Gray Level Zone Emphasis (HGLZE) |
| | 5 | Long Run Emphasis (LRE) | | 5 | Large Area Emphasis (LAE) |
| | 6 | Long Run High Gray Level Emphasis (LRHGLE) | | 6 | Large Area High Gray Level Emphasis (LAHGLE) |
| | 7 | Long Run Low Gray Level Emphasis (LRLGLE) | | 7 | Large Area Low Gray Level Emphasis (LALGLE) |
| | 8 | Low Gray Level Run Emphasis (LGLRE) | | 8 | Low Gray Level Zone Emphasis (LGLZE) |
| | 9 | Run Entropy (RE) | | 9 | Size Zone Non-Uniformity (SZNU) |
| | 10 | Run Length Non-Uniformity (RLNU) | | 10 | Size Zone Non-Uniformity Normalized (SZNUN) |
| | 11 | Run Length Non-Uniformity Normalized (RLNUN) | | 11 | Small Area Emphasis (LAE) |
| | 12 | Run Percentage (RP) | | 12 | Small Area High Gray Level Emphasis (SAHGLE) |
| | 13 | Run Variance (RV) | | 13 | Small Area Low Gray Level Emphasis (SALGLE) |
| | 14 | Short Run Emphasis (SRE) | | 14 | Zone Entropy (ZE) |
| | 15 | Short Run High Gray Level Emphasis (SRHGLE) | | 15 | Zone Percentage (ZP) |
| | 16 | Short Run Low Gray Level Emphasis (SRLGLE) | | 16 | Zone Variance (ZV) |
| First order (FO) | 1 | 10th Percentile | Gray-Level Cooccurrence Matrix (GLCM) | 1 | Autocorrelation |
| | 2 | 90th Percentile | | 2 | Cluster Prominence (CP) |
| | 3 | Energy | | 3 | Cluster Shade (CS) |
| | 4 | Entropy | | 4 | Cluster Tendency (CT) |
| | 5 | Interquartile Range (IQR) | | 5 | Contrast |
| | 6 | Kurtosis | | 6 | Correlation |
| | 7 | Maximum | | 7 | Difference Average (DA) |
| | 8 | Mean Absolute Deviation (MAD) | | 8 | Difference Entropy (DE) |
| | 9 | Mean | | 9 | Difference Variance (DV) |
| | 10 | Median | | 10 | Inverse Difference (ID) |
| | 11 | Minimum | | 11 | Inverse Difference Moment (IDM) |
| | 12 | Range | | 12 | Inverse Difference Moment Normalized (IDMN) |
| | 13 | Robust Mean Absolute Deviation (RMAD) | | 13 | Inverse Difference Normalized (IDN) |
| | 14 | Root Mean Squared (RMS) | | 14 | Informational Measure of Correlation 1 (IMC1) |
| | 15 | Skewness | | 15 | Informational Measure of Correlation 2 (IMC2) |
| | 16 | Total Energy (TE) | | 16 | Inverse Variance (IV) |
| | 17 | Uniformity | | 17 | Joint Average (JA) |
| | 18 | Variance | | 18 | Joint Energy (JEnergy) |
| Gray Level Dependence Matrix (GLDM) | 1 | Dependence Entropy (DE) | | 19 | Joint Entropy (JEntropy) |
| | 2 | Dependence Non-Uniformity (DNU) | | 20 | Maximal Correlation Coefficient (MCC) |
| | 3 | Dependence Non-Uniformity Normalized (DNUN) | | 21 | Maximum Probability (MP) |
| | 4 | Dependence Variance (DV) | | 22 | Sum Average (SA) |
| | 5 | Gray Level Non-Uniformity (GLNU) | | 23 | Sum Entropy (SE) |
| | 6 | Gray Level Variance (GLV) | | 24 | Sum of Squares (SS) |
| | 7 | High Gray Level Emphasis (HGLE) | Neighborhood Gray-Tone Difference Matrix (NGTDM) | 1 | Busyness |
| | 8 | Large Dependence Emphasis (LDE) | | 2 | Coarseness |
| | 9 | Large Dependence High Gray Level Emphasis (LDHGLE) | | 3 | Complexity |
| | 10 | Large Dependence Low Gray Level Emphasis (LDLGLE) | | 4 | Contrast |
| | 11 | Low Gray Level Emphasis (LGLE) | | 5 | Strength |
| | 12 | Small Dependence Emphasis (SDE) | | | |
| | 13 | Small Dependence High Gray Level Emphasis (SDHGLE) | | | |
| | 14 | Small Dependence Low Gray Level Emphasis (SDLGLE) | | | |
| | | | Shape 3D | 1 | Elongation |
| | | | | 2 | Flatness |
| | | | | 3 | Sphericity |
| | | | | 4 | Surface Area |
| | | | | 5 | Voxel Volume |
| | | | | 6 | Maximum 2D Diameter Row |
| | | | | 7 | Maximum 2D Diameter Slice |
| | | | | 8 | Maximum 3D Diameter |
| | | | | 9 | Least Axis Length |
| | | | | 10 | Surface Volume Ratio |
| | | | | 11 | Major Axis Length |
| | | | | 12 | Maximum 2D Diameter Column |
| | | | | 13 | Minor Axis Length |
| | | | | 14 | Mesh Volume |

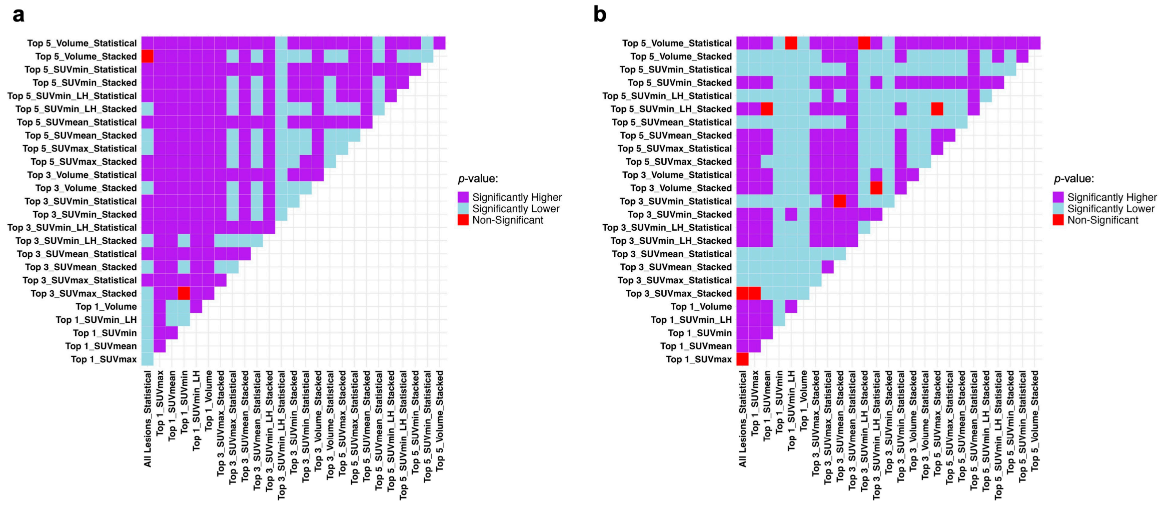


**Figure S1.** Mann-Whitney U test results comparing the average machine performance trained on all the datasets and sorting approaches used in progression event prediction regarding (a) recall and (b) specificity. It compares row (Model A) vs. column (Model B): purple = A significantly better, light blue = A significantly worse, red = No difference. SUV: Standardized Uptake Value, LH: Low-to-High.

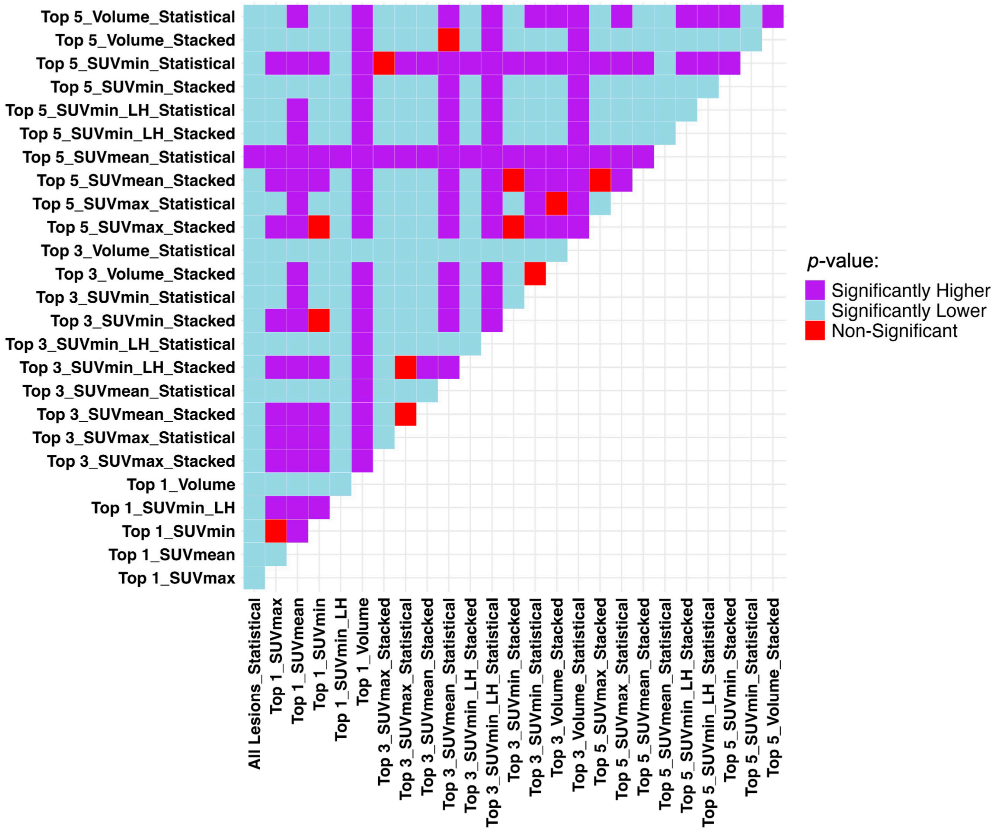


**Figure S2**. Mann-Whitney U test results comparing the average machine performance trained on all the datasets and sorting approaches used in time to progression (TTP) prediction regarding C-index. It compares row (Model A) vs. column (Model B): purple = A significantly better, light blue = A significantly worse, red = No difference. SUV: Standardized Uptake Value, LH: Low-to-High.

# METRICS Tool v1.0

**Please fill out all conditions first for relevant sections and then all active items to calculate METRICS score.**

**Please note that default option is "No".**

? Stands for explanation of items and conditions.

C Stands for conditional items or sections.

| Items/Conditions | Definitions | Weights | Options |
|---|---|---|---|
| **Study Design** | | | |
| Item#1 | ? Adherence to radiomics and/or machine learning-specific checklists or guidelines | 0.0368 | ● Yes ○ No |
| Item#2 | ? Eligibility criteria that describe a representative study population | 0.0735 | ● Yes ○ No |
| Item#3 | ? High-quality reference standard with a clear definition | 0.0919 | ● Yes ○ No |
| **Imaging Data** | | | |
| Item#4 | ? Multi-center | 0.0438 | ○ Yes ● No |
| Item#5 | ? Clinical translatability of the imaging data source for radiomics analysis | 0.0292 | ● Yes ○ No |
| Item#6 | ? Imaging protocol with acquisition parameters | 0.0438 | ○ Yes ● No |
| Item#7 | ? The interval between imaging used and reference standard | 0.0292 | ● Yes ○ No |
| **Segmentation** C | | | |
| Condition#1 | ? Does the study include segmentation? | | ● Yes ○ No |
| Condition#2 | ? Does the study include fully automated segmentation? | | ○ Yes ● No |
| Item#8 | ? Transparent description of segmentation methodology | 0.0337 | ● Yes ○ No |
| Item#9 | ? Formal evaluation of fully automated segmentation C | 0.0225 | ○ Yes ○ No |
| Item#10 | ? Test set segmentation masks produced by a single reader or automated tool | 0.0112 | ○ Yes ● No |
| **Image Processing and Feature Extraction** | | | |
| Condition#3 | ? Does the study include hand-crafted feature extraction? | | ● Yes ○ No |
| Item#11 | ? Appropriate use of image preprocessing techniques with transparent description | 0.0622 | ● Yes ○ No |
| Item#12 | ? Use of standardized feature extraction software C | 0.0311 | ● Yes ○ No |
| Item#13 | ? Transparent reporting of feature extraction parameters, otherwise providing a default configuration statement | 0.0415 | ● Yes ○ No |
| **Feature Processing** | | | |
| Condition#4 | ? Does the study include tabular data? | | ● Yes ○ No |
| Condition#5 | ? Does the study include end-to-end deep learning? | | ○ Yes ● No |
| Item#14 | ? Removal of non-robust features C | 0.0200 | ○ Yes ● No |
| Item#15 | ? Removal of redundant features C | 0.0200 | ● Yes ○ No |
| Item#16 | ? Appropriateness of dimensionality compared to data size C | 0.0300 | ● Yes ○ No |
| Item#17 | ? Robustness assessment of end-to-end deep learning pipelines C | 0.0200 | ○ Yes ○ No |
| **Preparation for Modeling** | | | |
| Item#18 | ? Proper data partitioning process | 0.0599 | ● Yes ○ No |
| Item#19 | ? Handling of confounding factors | 0.0300 | ○ Yes ● No |
| **Metrics and Comparison** | | | |



**Figure S3.** First page of METRICS checklist.

| | | | | |
|---|---|---|---|---|
| Item#20 | ? | Use of appropriate performance evaluation metrics for task | 0.0352 | ◉ Yes ○ No |
| Item#21 | ? | Consideration of uncertainty | 0.0234 | ◉ Yes ○ No |
| Item#22 | ? | Calibration assessment | 0.0176 | ○ Yes ◉ No |
| Item#23 | ? | Use of uni-parametric imaging or proof of its inferiority | 0.0117 | ○ Yes ◉ No |
| Item#24 | ? | Comparison with a non-radiomic approach or proof of added clinical value | 0.0293 | ○ Yes ◉ No |
| Item#25 | ? | Comparison with simple or classical statistical models | 0.0176 | ◉ Yes ○ No |
| **Testing** | | | | |
| Item#26 | ? | Internal testing | 0.0375 | ◉ Yes ○ No |
| Item#27 | ? | External testing | 0.0749 | ○ Yes ◉ No |
| **Open Science** | | | | |
| Item#28 | ? | Data availability | 0.0075 | ○ Yes ◉ No |
| Item#29 | ? | Code availability | 0.0075 | ○ Yes ◉ No |
| Item#30 | ? | Model availability | 0.0075 | ○ Yes ◉ No |
| | | | **Total METRICS score:** | **68.2%** |
| | | | ? **Quality category:** | **Good** |
| | | | ? **Publication ID:** | |

**If you publish any work which uses this tool, please cite the following publication:**

Kocak B, Akinci D'Antonoli T, Mercaldo N, et al. METhodological RadiomICs Score (METRICS): a quality scoring tool for radiomics research endorsed by EuSoMII. Insights Imaging. 2024;15(1):8. Published 2024 Jan 17. doi:10.1186/s13244-023-01572-w



**Figure S4.** Second page of METRICS checklist.
